# Transmission dynamics and control of *Rickettsia rickettsii* in populations of *Hydrochoerus hydrochaeris* and *Amblyomma sculptum*

**DOI:** 10.1371/journal.pntd.0005613

**Published:** 2017-06-05

**Authors:** Gina Polo, Carlos Mera Acosta, Marcelo B. Labruna, Fernando Ferreira

**Affiliations:** 1 Department of Preventive Veterinary Medicine and Animal Health, Faculty of Veterinary Medicine, University of São Paulo, São Paulo, SP, Brazil; 2 Institute of Physics. University of São Paulo, São Paulo, SP, Brazil; University of California Davis, UNITED STATES

## Abstract

**Background:**

Brazilian Spotted Fever (BSF), caused by the bacterium *Rickettsia rickettsii*, is the tick-borne disease that generates the largest number of human deaths in the world. In Brazil, the current increase of BSF human cases has been associated with the presence and expansion of capybaras *Hydrochoerus hydrochaeris*, which act as primary hosts for the tick *Amblyomma sculptum*, vector of the *R. rickettsii* in this area.

**Methods:**

We proposed a semi-discrete-time stochastic model to evaluate the role of capybaras in the transmission dynamics of *R. rickettsii*. Through a sensitivity analysis, we identified the parameters with significant influence on the *R. rickettsii* establishment. Afterward, we implemented the Gillespie’s algorithm to simulate the impact of potential public health interventions to prevent BSF human cases.

**Results:**

The introduction of a single infected capybara with at least one infected attached tick is enough to trigger the disease in a non-endemic area. We found that to avoid the formation of new BSF-endemic areas, it is crucial to impede the emigration of capybaras from endemic areas by reducing their birth rate by more than 58%. Model results were corroborated by *ex-situ* data generated from field studies, and this supports our proposal to prevent BSF human cases by implementing control strategies focused on capybaras.

**Conclusion:**

The proposed stochastic model illustrates how strategies for the control and prevention of vector-borne infectious diseases can be focused on amplifier hosts management practices. This work provides a basis for future prevention strategies for other neglected vector-borne diseases.

## Author Summary

A stochastic model for the spread of *R. rickettsii* among the *Amblyomma sculptum* tick vector and the *Hydrochoerus hydrochaeris*, amplifier host, was formulated. We found that the introduction of a single infected capybara, with at least one infected attached tick, is enough to trigger the disease in a non-endemic area. To avoid disease propagation, it is crucial to impede the emigration of capybaras from endemic areas by keeping the population in a stable state. Otherwise, to eliminate the disease from endemic areas, a reduction by more than 58% in the capybara birth rate is necessary. These results allow improving the planning of public actions to prevent Brazilian Spotted fever based on the control of capybara population and provided a basis for planning prevention programs for other neglected vector-borne diseases.

## Introduction

*Rickettsia rickettsii* is the etiological agent of the Brazilian spotted fever (BSF), the deadliest spotted fever in the world. This infection is partially pathogenic to *Amblyomma sculptum* ticks, main vectors of the *R. rickettsii* in South America [1, 2], generating a drop in the infection rate with each tick generation [3]. In addition, *A. sculptum* ticks are unable to maintain the *R. rickettsii* infection in successive generations by transovarial and transstadial transmissions [4]. Hence, the maintenance of *R. rickettsii* depends on a constant introduction of new susceptible animals (i.e., newborn of vertebrate hosts), which act as amplifier hosts and guarantee the constant creation of new cohorts of infected ticks [4–7]. This suggests that control strategies focused on vectors [8, 9] are not enough for the prevention of this tick-borne infectious disease.

In Brazil, the capybara *Hydrochoerus hydrochaeris* acts as the amplifier host of *R. rickettsii* infection [10, 11]. In southeastern Brazil, both capybaras and BSF occurrences have increased significantly over the last three decades [5, 12]. In turn, these occurrences have been spatiotemporally associated with rising production and spatial expansion of sugarcane crops, the main food source of capybaras [13]. In BSF-endemic areas, population densities of capybaras have reached numbers up to 40 times higher than those recorded in natural environments such as the Amazon and Pantanal [14]. However, the effectiveness of control strategies focused on this amplifier host and their impact on the transmission of *R. rickettsii* are unknown.

The dynamics of complex transmission cycles, such as tick-borne infectious diseases have been broadly analyzed. Hudson et al. published the first deterministic model representing the dynamics of the Louping-ill disease, an acute viral zoonosis which mainly affects sheep [15]. O’Callaghan et al. put forward tick-borne diseases dynamics considering the potential effect of a vaccination program for *Ehrlichia ruminantium* [16]. Similarly, Rosà et al. developed a model for the tick-borne encephalitis virus including the tick stages and different transmission routes [17, 18]. Despite their important role in the understanding of vector-borne diseases dynamics, unfortunately they do not include the seasonal behavior of the vector life cycle, neither the onset nor the extinction of these diseases. As it is well established, stochastic models should be used in phenomena that do not satisfy the law of large numbers such as large communities with minor outbreaks [19]. In fact, the extinction of endemic diseases can only be analyzed with stochastic models, since extinction occurs when the epidemic process deviates from the expected level [19].

In this work, we propose a discrete-state semi-discrete-time stochastic framework to evaluate the role of capybaras in the transmission dynamics of *R. rickettsii*. We identify the parameters with significant influence on the *R. rickettsii* establishment and subsequently evaluate the impact of potential public health interventions to prevent BSF in humans.

## Materials and methods

### Model

**Fig 1:**
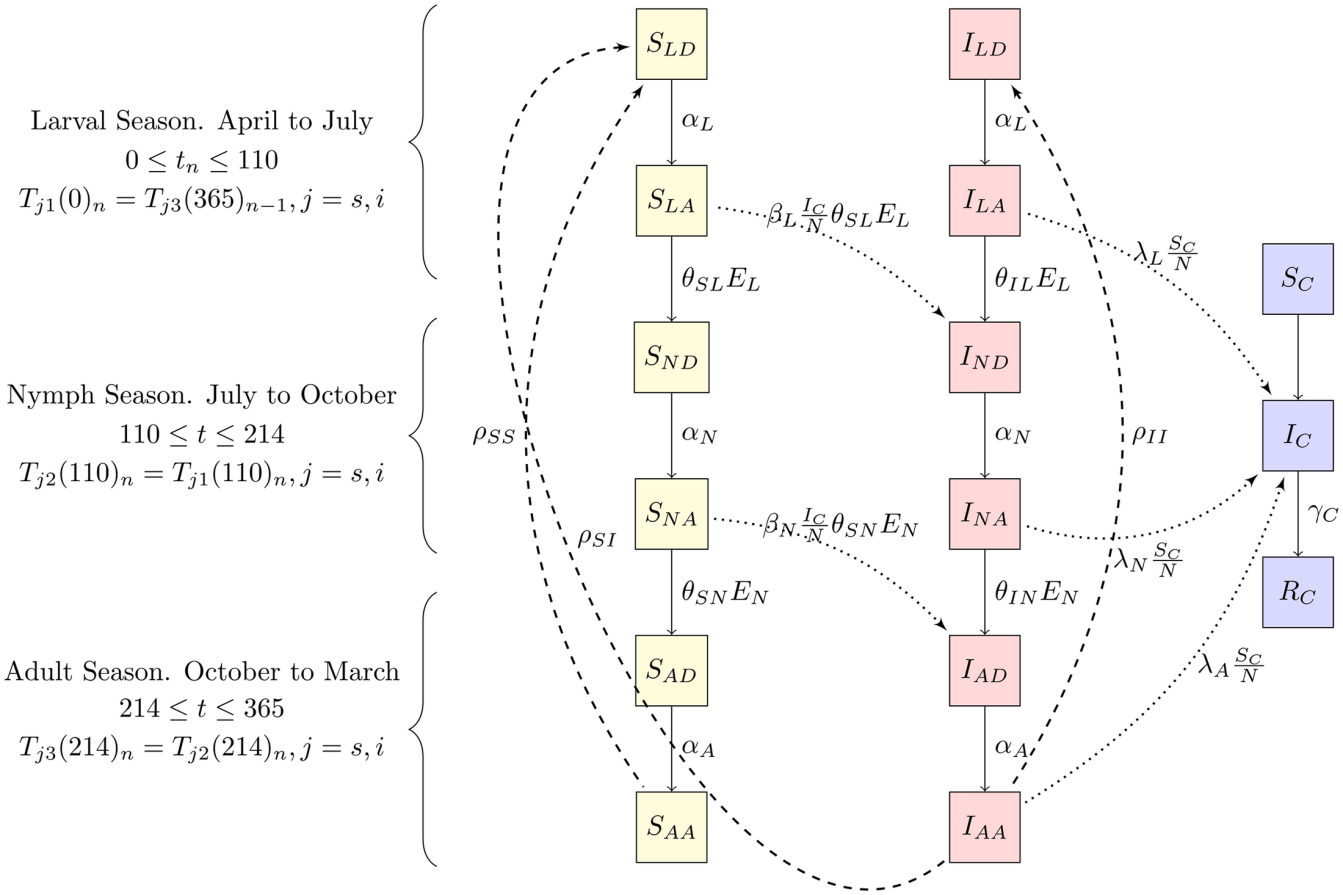
Schematic representation of the *R. rickettsii* dynamics in populations of *H. hydrochaeris* and *A. sculptum*. Deaths, births, and migrations are not represented in the capybara population. Tick deaths are also not represented.

The *R. rickettsii* dynamics in populations of *H. hydrochaeris* and *A. sculptum* is represented in Fig 1. Our model was adjusted to a semi-discrete time dynamics in order to consider the 1-year life cycle of the tick *A. sculptum*, which is primarily controlled by the larval behavioral diapause [20]. Thus, larvae exclusively quest and feed from April to July for 110 days, nymphs from July to October for 104 days and adults particularly quest, feed and reproduce from October to March for 151 days, as shown in Fig 1.

The tick population is classified according to the life cycle stages as larvae (L), nymphs (N) or adults (A), which could be detached from a capybara or attached to it. When a tick gets infected by an infected capybara, it remains infected until it dies. Thus, each *A. sculptum* stage is also classified according to whether it is susceptible (S) or infected (I). In this way, the population of each tick stage is represented by three indexes, where the first index denotes the infection status, the second index denotes the tick life cycle stage, and the third index denotes detachment (D) or attachment (A).

Attached ticks at the larval and nymph stages detach at the respective rates of *θ*_*L*_ and *θ*_*N*_. Furthermore, detached ticks at larval, nymph and adult stages can attach at rates *α*_*L*_, *α*_*N*_ and *α*_*A*_, and can die at rates *δ*_*L*_, *δ*_*N*_ and *δ*_*A*_. The production rate *ρ* of larvae is assumed to be proportional to the total number of adult attached ticks *AA*.

On the other hand, capybaras are classified as susceptible *S*_*C*_, infected *I*_*C*_ and recovered *R*_*C*_, as shown in Fig 1. They reproduce at a constant rate *μ*_*C*_, die at rate *δ*_*C*_ and recovered at rate *γ*. All capybaras have the same susceptibility and there is no increased death rate of infected individuals due to disease. Susceptible capybaras can get infected by an attached larva, nymph or adult tick at a rate of λ_*L*_, λ_*N*_ and λ_*A*_, respectively. Once capybaras are infected, they keep the *R. rickettsii* in the bloodstream for 7 to 10 days [11], during which the infection of new susceptible larvae or nymphs that feed on it can occur at rates *β*_*L*_ and *β*_*N*_, respectively. After this period, capybaras become immune to the disease. We do not consider a transmission rate from infected capybaras to susceptible adult ticks *β*_*A*_, because of the time of *R. rickettsii* infection in ticks is greater than the time of laying. Thus, eggs can be infected only if adult ticks become infected during the larval or nymph stages [4]. Additionally, we consider a vertical transmission and hence infected adult ticks can produce infected detached larvae at rate *ρ*_*II*_. In this way, there are 25 distinct reactions in the stochastic model which are listed in Table 1. The equivalent deterministic equations associated with the stochastic reactions are presented in the S1 File.

**Table 1:** Events and reactions of the tick-capybara-disease stochastic process.

Event	Reaction
Birth of capybara	$S_{C}\overset{\mu_{C}N}{\to }S_{C}+1$
Birth of susceptible detached larvae	$S_{LD}\overset{\rho_{SS}S_{AA}+\rho_{SI}I_{AA}}{\to }S_{LD}+1$
Birth of infected detached larvae	$I_{LD}\overset{\rho_{II}I_{AA}}{\to }I_{LD}+1$
Engorgement of a susceptible larva	$S_{LD}\overset{\alpha_{L}S_{LD}}{\to }\left(S_{LD}-1\right)+\left(S_{LA}+1\right)$
Engorgement of an infected larva	$I_{LD}\overset{\alpha_{L}I_{LD}}{\to }\left(I_{LD}-1\right)+\left(I_{LA}+1\right)$
Transmission from an infected capybara to a susceptible larvae	$S_{LA}\overset{\beta_{L}\frac{I_{C}}{N}\theta_{SL}E_{L}S_{LA}}{\to }\left(S_{LA}-1\right)+\left(I_{ND}+1\right)$
Transmission from an infected larvae to a susceptible capybara	$S_{C}\overset{\lambda_{L}\frac{S_{C}}{N}I_{LA}}{\to }\left(S_{C}-1\right)+\left(I_{C}+1\right)$
Stage change from susceptible larvae to detached nymph	$S_{LA}\overset{\theta_{S_{L}}E_{L}S_{LA}}{\to }\left(S_{LA}-1\right)+\left(S_{ND}+1\right)$
Stage change from infected larvae to detached nymph	$S_{LA}\overset{\theta_{I_{L}}E_{L}I_{LA}}{\to }\left(S_{LA}-1\right)+\left(S_{ND}+1\right)$
Recovery rate of capybara	$I_{C}\overset{\gamma_{C}I_{C}}{\to }\left(I_{C}-1\right)+\left(R_{C}+1\right)$
Death of a susceptible capybara	$S_{C}\overset{\delta_{C}S_{C}}{\to }S_{C}-1$
Emigration of a susceptible capybara	$S_{C}\overset{\epsilon_{C}S_{C}}{\to }S_{C}-1$
Death of an infected capybara	$I_{C}\overset{\delta_{C}I_{C}}{\to }I_{C}-1$
Emigration of an infected capybara	$I_{C}\overset{\epsilon_{C}I_{C}}{\to }I_{C}-1$
Death of a recovered capybara	$R_{C}\overset{\delta_{C}R_{C}}{\to }R_{C}-1$
Emigration of a recovered capybara	$R_{C}\overset{\epsilon_{C}R_{C}}{\to }R_{C}-1$
Engorgement rate of a susceptible nymph	$S_{ND}\overset{\alpha_{N}S_{ND}}{\to }\left(S_{ND}-1\right)+\left(S_{NA}+1\right)$
Engorgement rate of an infected nymph	$I_{ND}\overset{\alpha_{N}I_{ND}}{\to }\left(I_{ND}-1\right)+\left(I_{NA}+1\right)$
Transmission from an infected nymph to a susceptible capybara	$S_{C}\overset{\lambda_{N}\frac{S_{C}}{N}I_{NA}}{\to }\left(S_{C}-1\right)+\left(I_{C}+1\right)$
Transmission from an infected capybara to a susceptible nymph	$S_{NA}\overset{\beta_{N}\frac{I_{C}}{N}\theta_{SN}E_{N}S_{NA}}{\to }\left(S_{NA}-1\right)+\left(I_{AD}+1\right)$
Stage change from susceptible nymph to detached adult	$S_{NA}\overset{\theta_{S_{N}}E_{N}S_{NA}}{\to }\left(S_{NA}-1\right)+\left(S_{AD}+1\right)$
Stage change from infected nymph to detached adult	$I_{NA}\overset{\theta_{I_{N}}E_{N}I_{NA}}{\to }\left(I_{NA}-1\right)+\left(I_{AD}+1\right)$
Engorgement of a susceptible adult	$S_{AD}\overset{\alpha_{A}S_{AD}}{\to }\left(S_{AD}-1\right)+\left(S_{AA}+1\right)$
Engorgement of an infected adult	$I_{AD}\overset{\alpha_{A}I_{AD}}{\to }\left(I_{AD}-1\right)+\left(I_{AA}+1\right)$
Transmission from an infected adult tick to a susceptible capybara	$S_{C}\overset{\lambda_{A}\frac{S_{C}}{N}I_{AA}}{\to }\left(S_{C}-1\right)+\left(I_{C}+1\right)$

### Time series simulations

The stochastic process detailed in Table 1 was simulated in the R programming language employing the Gillespie’s algorithm. For each simulation we performed the number of iterations needed to get a stable performance.

We assumed a finite number of individuals distributed over a finite set of discrete states. Given an initial time *t*_0_ and an initial population state *X*(*t*_0_), the Gillespie’s algorithm allows us to generate the time-evolution trajectory of the state vector *X*(*t*) ≡ (*X*_1_(*t*), …, *X*_*N*_(*t*)), where *X*_*i*_(*t*) is the population size of state *i* at time *t* and *N* is the number of states. Changes in the number of individuals in each state occur due to reactions between interacting states. The states interact through *M* reactions *R*_*j*_, where *j* = 1, …, *M* denotes the *j*th reaction. A reaction is defined as any process that instantaneously changes the population size of at least one state [21, 22]. The time step to the next reaction was then determined as $\tau =\frac{1}{\alpha_{0}\left(x\right)}ln\left(1/r_{1}\right)$, where *α*_0_(*x*) = Σ*α*_*j*_(*x*) and the index of the next reaction to execute *R*_*j*_ is the smallest integer *j* satisfying $j=\Sigma_{i=1}^{j}\alpha_{i}\left(x\right)>r_{2}\alpha_{0}\left(x\right)$ [21, 22].

All parameters were estimated using previously generated data from *ex situ* field works in southeastern Brazil [4, 5, 11, 14, 23]. It is noteworthy that the natural capybara birth rate *μ* assumes the value of 70% of adults, 64% of females, a litter size mean of 4.2 pups, 1.23 births per female per year and a pregnancy success of 85% [14, 23]. If capybaras die at an exponential rate, then *δ*_*C*_ is the fraction required to die each day. The birth rate of a susceptible tick was determined assuming a female weight of 500mg [4], CEI (mg egg mass/mg engorged female × 100) of 48.4% [4],18.8 eggs/ 1 mg of eggs [24] and hatching success of 68% [4]. Likewise, the birth rate of an infected tick was determined assuming a transovarial transmission of 42.8% [4], filial infection rate of 50% [4], female weight of 372.20 mg [4], CEI of 39.55% [4], 18.8 eggs/1 mg of eggs, [24] and hatching success of 44.2% [4]. We consider a population of 20 adult female ticks per capybara and the groups of capybaras were restricted to 50 individuals [14, 23, 25]. A full list of the model’s parameters used in the simulations is given in Table 2.

**Table 2:** Parameters and values used in simulations.

Param.	Value	Description
*μ*	0.005 *d*^−1^	Birth rate of capybara [14, 23]
*ρ*_*SS*_	2709%	Susceptible larvae production per adult susceptible tick [4]
*ρ*_*SI*_	305%	Susceptible larvae production per adult infected tick [4]
*ρ*_*II*_	228%	Infected larvae production per adult infected tick [4]
*E*_*L*_	10%	Larval engorgement [4]
*α*_*L*_	0.003 *d*^−1^	Attached rate of a larva [4]
*θ*_*SL*_	35%	Stage change susceptible larvae [4]
*θ*_*IL*_	17%	Stage change infected larvae [4]
*α*_*N*_	0.006 *d*^−1^	Attached rate of a nymph [4]
*E*_*N*_	40%	Nymph engorgement [4]
*θ*_*SN*_	60%	Stage change susceptible nymph [4]
*θ*_*IN*_	60%	Stage change infected nymph [4]
*α*_*A*_	0.009 *d*^−1^	Attached rate of an adult [4]
*E*_*A*_	70%	Adult engorgement [4]
λ_*L*_	9.4×10^−5^ *d*^−1^	Transmission from an infected larvae to a susceptible capybara [11]
λ_*N*_	0.046 *d*^−1^	Transmission from an infected nymph to a susceptible capybara [11]
λ_*A*_	0.046 *d*^−1^	Transmission from an infected adult tick to a susceptible capybara [11]
*β*_*L*_	12%	Transmission from an infected capybara to a susceptible larvae [4, 11]
*β*_*N*_	25%	Transmission from an infected capybara to a susceptible nymph [4, 11]
(1/*γ*)	10 days	Capybara’s infecion period [5]
*γ*	0.027 *d*^−1^	Recovery rate of capybaras [5]
*δ*_*C*_	0.002 *d*^−1^	Death rate of capybaras [14]
*ϵ*_*C*_	0.003 *d*^−1^	Emigration rate of capybaras

### Sensitivity analysis

To quantify the impact of the variation of each parameter on the output of the BSF model, we combined uncertainty through the Latin hypercube sampling (LHS) with the robust Partial rank correlation coefficient (PRCC) method [26, 27]. Initially, we obtained a random parameter distribution divided into one hundred equal probability intervals, which were then sampled. Thus, a LHS matrix was generated with one hundred rows for the number of simulations (sample size) and six columns corresponding to the number of varied parameters (*α*, *μ*_*C*_, λ, *γ*_*C*_, *δ*_*C*_, *ϵ*_*C*_). The parameters *α* and λ were varied according to the life cycle season. BSF model solutions were then simulated using each combination of parameter values through ten year simulation. One thousand model outputs were obtained and the parameter and output values were transformed into their ranks. Subsequently, we computed the PRCCs between each parameter and the average infected population size.

## Results and discussion

**Fig 2:**
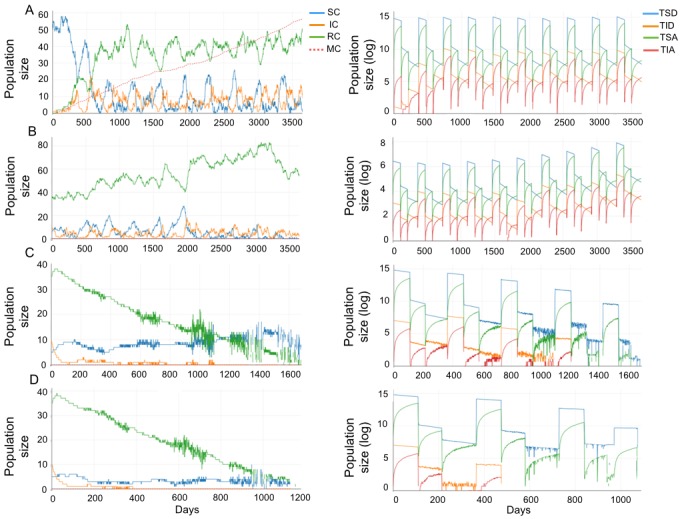
Simulations of *R. rickettsii* dynamics in populations of *H. hydrochaeris* (left) and *A. sculptum* (right) in different scenarios. (A) Introduction of a single infected capybara with an infected attached tick. Initial values correspond with *μ* = 0.0054, *S*_*C*_ = 49, *I*_*C*_ = 1, *R*_*C*_ = 0, *S*_*AA*_ = 1000 and *I*_*AA*_ = 1. Migratory capybaras (MC) values represent one-tenth of the real value. (B) Endemic area with a stable state of capybaras, ensuring no emigration. Initial values correspond with *μ* = 0.0021, *S*_*C*_ = 5, *I*_*C*_ = 10, *R*_*C*_ = 35, *S*_*AA*_ = 1000 and *I*_*AA*_ = 5. (C) Endemic area with a decrease of 80% and (D) 90% in the birth rate of capybaras. Oscillations correspond with the seasonality behavior of *R. rickettsii* dynamics.

Initially, we simulated the formation of an endemic area by the introduction of infected capybaras and infected attached ticks. For a more realistic simulation of current BSF-endemic areas of southeastern Brazil, we consider a growing population of capybaras (births greater than deaths and high emigration). We assume that this scenario corresponds with the onset of a BSF epidemic. We found that the introduction of a single infected capybara or a single infected tick is unable to trigger the disease in a non-endemic area of 50 susceptible capybaras. However, the introduction of an infected capybara with at least one infected tick attached is enough to establish a new endemic area, as shown in Fig 2 scenario A. This scenario illustrates how the fraction of infected capybaras and ticks converges to a constant from year 2 onward. In this equilibrium state, the average fraction of infected capybaras is 8.9% (95% CI = 0%-28.6%), 18.2% (95% CI = 0%-44.9%) and 17.5% (95% CI = 0%-43.9%) in the larvae, nymphs and adults season respectively. In this scenario, the average fraction of infected detached larvae is 1.25% (95% CI = 0%-7.04%), infected attached larvae 0.7% (95% CI = 0%-6.5%), infected detached nymphs 1.35% (95% CI = 0%-9.36%), infected attached nymphs 0.8% (95% CI = 0%-6.95%), infected detached adults 0.46% (95% CI = 0%-5.17%) and infected attached adults 0.54% (95% CI = 0%-5.64%). These results are consistent with previous observations in BSF-endemic areas in which the fraction of infected *A. sculptum* adults attached to horses has been reported at 0% [28] and the fraction of infected detached adults *A. sculptum* at 1% (95% CI = 0.01%-7.8%) [28], 0.2% (95% CI = 0.01%-1.04%) [10] and 1.28% (95% CI = 0.07%-5.59%) [29]. The fraction of infected capybaras and other tick populations remains unreported.

In scenario A, an average of 57.1% (95% CI = 22.8%-91.4%), 78.2% (95% CI = 49.6%-99.9%), and 77.2% (95% CI = 48.1%-99.9%) of capybaras became immune after a primary infection with *R. rickettsii* in the larvae, nymph and adult seasons, respectively. In practical terms, these immune capybaras include the *Rickettsia*-seropositive animals that are usually found in serosurvey studies in BSF-endemic areas. These numbers agree with previous serosurvey studies that reported 50-80% of capybaras to be seropositive for *R. rickettsii* in BSF-endemic areas [10, 30]. In this established endemic area, we observe that 563 capybaras migrated over 10 years.

**Fig 3:**
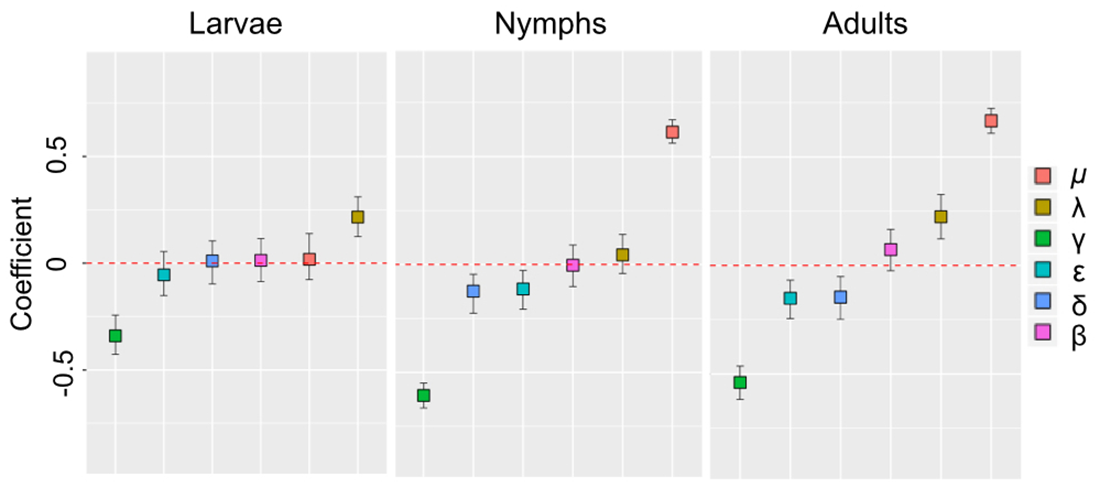
Partial rank correlation coefficient (PRCC) between each parameter and average infected population after 10 years in each tick life-cycle season. Variations in the birth rate and in the recovery rate of capybaras have the greatest effect on the *R. rickettsii* maintenance in the nymphs and adult tick seasons. Parameters with negative PRCC will decrease the number of infected individuals as they increased.

Sensitivity analysis of scenario A show that the capybaras birth rate variation had the greatest impact on the average infected population size. In the nymph and adult ticks seasons, when the infection of capybaras is greater, the correlation value between the birth rate of capybaras and the average infected population is PRCC>0.6, being significant in both seasons (Fig 3). Since in BSF-endemic areas of southeastern Brazil the population of capybaras is growing [5, 12–14], theoretically, the epidemic will survive forever [31]. Considering *μ* = *δ*_*C*_ + *ϵ*, where *μ*_*C*_ represents birth, *δ*_*C*_ death and *ϵ* emigration rate of capybaras, the population growth generates a high rate of emigration and consequently the spread of the disease. In this way, to better understand the effect of changes in capybara population, we investigate the host-tick-infection dynamics for different values of *μ*_*C*_, under three additional scenarios: 1) an endemic area with a stable state of capybaras (births equal deaths, no emigration and no importation of the disease from outside); 2) an endemic area with a decrease of 80% and 3) 90% in the birth rate of capybaras.

Contrary to the expected behavior [32], when the population is in a stable state, the disease does not disappear. Indeed, the proportion of infected individuals remains constant over time from year 2 onward as shown in Fig 2B. In this scenario, the average fraction of infected capybaras is 4.6% (95% CI = 0.01%-19.3%), 6.3% (95% CI = 0.01%-23.1%) and 7.4% (95% CI = 0.01%-25.4%) in the larvae, nymphs and adults seasons, respectively. To ensure births equals deaths (*μ*_*C*_ = *δ*_*C*_) and thereby guarantee no emigration (*ϵ* = 0), it is necessary to control the capybara’s birth rate in 58%. Thereby, though the disease does not disappear, this birth rate guarantees that the *R. rickettsii* will not spread from BSF-endemic areas.

Thus, for the elimination of the *R. rickettsii* from endemic areas, a decrease in the capybara’s birth rate to values lower than 0.0021 is necessary. However, this can lead to a decline in capybaras population over time. When a decrease of 80% (*μ*_*C*_ = 0.0011) in the birth rate of capybaras is considered, infected individuals tend to disappear in the fourth year along with a decrease in the total population size, as shown in Fig 2C. Otherwise, when a decrease of of 90% (*μ*_*C*_ = 0.0005) in the birth rate of capybaras is considered, infected ticks and capybaras tend to disappear from the second year as shown in Fig 2D.

Strategies to reduce the birth rate of capybaras include the reduction of the carrying capacity, their removal, either by euthanasia or regulated hunting, and their reproductive control. As capybaras natality depends primarily on the availability of food sources, as is typically the case for rodents [33], the reduction of the carrying capacity in BSF-endemic areas is a plausible strategy to reduce their birth rate [34]. Polo et al. found a spatiotemporal relationship between the occurrence of BSF and the increment and expansion of sugarcane crops, the main food source of capybaras in southeastern Brazil and the most important agricultural product in the region [13]. Furthermore, in this area, there is a constant availability of water sources, which allow the establishment of capybaras, as this is a semiaquatic vertebrate that depends on water sources for thermic regulation, reproduction, and predator protection [33]. Certainly, controlling these aspects is not feasible.

Additionally, because of the constant increment and abundance of vital resources offered by the environment in BSF-endemic areas of southeastern Brazil, it is important to consider that in response to the removal or elimination of recovering capybaras from theses areas, a reintroduction of susceptible animals can occur [13]. This, along with the long survival of unfed *A. sculptum* in pastures [35], and the fact that just one infected capybara with a single infected tick attached is sufficient to establish an endemic area, can cause a rapid spread of the disease and consequently an increased risk of transmission to humans.

Reproductive control of capybaras through deferentectomy and ligation of fallopian tubes was previously tested in southeastern Brazil [36]. It was observed that the reproductive management did not negatively influence individual or collective behavioral aspects, with the animals defending their territory and not migrating [36]. The sterilization of capybaras has already been authorized as a way to prevent BSF human cases in a small endemic area of southeastern Brazil [37].

Future studies encompassing field data should be performed to evaluate the role of alternative reservoirs in the dynamics of *R. rickettsii*. This is a limitation of our work since we considered BSF-endemic areas of southeastern Brazil, where capybaras are the major, though not the exclusive, hosts for either larval, nymphal or adult stages of *A. sculptum*. In most BSF-endemic areas, the only medium-to large-sized animal species is the capybara; therefore it is the only host for the *A. sculptum* adult stage, and consequently, the only suitable host species to sustain an *A. sculptum* population in the area [7, 10, 38]. However, there have been some previous reports of *A. sculptum* immature stages (larvae and nymphs) on small animals (wild mice, marsupials, birds) which usually share the same habitat with capybaras [39, 40]. Nevertheless, comparing to capybaras, the amount of larvae or nymphs that feed on these animals is minimal; i.e., while hundreds to thousands larvae and nymphs are commonly found feeding on a single capybara [41], we usually find less than 10, or exceptionally a few dozen *A. sculptum* ticks on individual small animals [39–41]. Indeed, this condition is also favored by the fact that all active stages of *A. sculptum* tend to host-quest on vegetation above 15 cm from the soil, precluding their direct contact with small mammals or ground-feeding passerine birds [20, 42].

This work offers an alternative for the planning of prevention strategies for tick-borne neglected infectious diseases. The planning of these type of interventions is usually performed by public entities through heuristic techniques, such as trial and error methods, without obtaining optimal results. The results of our work accurately match with data previously generated from field studies on the *R. rickettsii* dynamics in southeastern Brazil and will potentially allow the formulation of public policy to prevent BSF human cases based on the control of capybara population growth. Furthermore, this work will provide a basis for the planning of prevention programs for other neglected vector-borne infectious diseases.

## Supporting information

S1 File Equivalent deterministic equations associated with the reactions of the stochastic model for the larval, nymph and adult tick seasons. (PDF)
