# Supplementary material for: Transmission dynamics and control of Rickettsia rickettsii in populations of Hydrochoerus hydrochaeris and Amblyomma sculptum
Source: PLoS Negl Trop Dis. 2017 Jun 5;11(6):e0005613. doi: 10.1371/journal.pntd.0005613 (PMC5472331; doi:10.1371/journal.pntd.0005613)
Supplement: S1 File — (PDF) [file pntd.0005613.s001.pdf]

Supporting information S1

The equivalent deterministic equations associated with the reactions of the stochastic model for the larval season are:

$$\begin{aligned}
\frac{dS_{LD}}{dt} &= \rho_{SS}S_{AA} + \rho_{SI}I_{AA} - \alpha_L S_{LD} \\
\frac{dI_{LD}}{dt} &= \rho_{II}I_{AA} - \alpha_L I_{LD} \\
\frac{dS_{LA}}{dt} &= \alpha_L S_{LD} - \beta_L \frac{I_C}{N}\theta_{SL}E_L S_{LA} - \theta_{S_L}E_L S_{LA} \\
\frac{dI_{LA}}{dt} &= \alpha_L I_{LD} - \theta_{I_L}E_L I_{LA} \\
\frac{dS_C}{dt} &= \mu_C N - \lambda_L \frac{S_C}{N} I_{LA} - \delta_C S_C - \epsilon_C S_C \\
\frac{dI_C}{dt} &= \lambda_L \frac{S_C}{N} I_{LA} - \gamma_C I_C - \delta_C I_C - \epsilon_C I_C \\
\frac{dR_C}{dt} &= \gamma_C I_C - \delta_C R_C - \epsilon_C R_C
\end{aligned}$$

Similarly, the equivalent deterministic equations for the nymph season can be written as:

$$\begin{aligned}
\frac{dS_{ND}}{dt} &= \theta_{S_L}E_L S_{LA} - \alpha_N S_{ND} \\
\frac{dI_{ND}}{dt} &= \beta_L \frac{I_C}{N}\theta_{SL}E_L S_{LA} + \theta_{I_L}E_L I_{LA} - \alpha_N I_{ND} \\
\frac{dS_{NA}}{dt} &= \alpha_N S_{ND} - \beta_N \frac{I_C}{N}\theta_{SN}E_N S_{NA} - \theta_{S_N}E_N S_{NA} \\
\frac{dI_{NA}}{dt} &= \alpha_N I_{ND} - \theta_{I_N}E_N I_{NA} \\
\frac{dS_C}{dt} &= \mu_C N - \lambda_N \frac{S_C}{N} I_{NA} - \delta_C S_C - \epsilon_C S_C \\
\frac{dI_C}{dt} &= \lambda_N \frac{S_C}{N} I_{NA} - \gamma_C I_C - \delta_C I_C - \epsilon_C I_C \\
\frac{dR_C}{dt} &= \gamma_C I_C - \delta_C R_C - \epsilon_C R_C
\end{aligned}$$

Finally, the equivalent deterministic equations for the adult season are

$$\frac{dS_{AD}}{dt} = \theta_{S_N} E_N S_{NA} - \alpha_A S_{AD}$$
$$\frac{dI_{AD}}{dt} = \beta_N \frac{I_C}{N} \theta_{SN} E_N S_{NA} + \theta_{I_N} E_N I_{NA} - \alpha_A I_{AD}$$
$$\frac{dS_{AA}}{dt} = \alpha_A S_{AD}$$
$$\frac{dI_{AA}}{dt} = \alpha_A I_{AD}$$
$$\frac{dS_C}{dt} = \mu_C N - \lambda_A \frac{S_C}{N} I_{AA} - \delta_C S_C - \epsilon_C S_C$$
$$\frac{dI_C}{dt} = \lambda_A \frac{S_C}{N} I_{AA} - \gamma_C I_C - \delta_C I_C - \epsilon_C I_C$$
$$\frac{dR_C}{dt} = \gamma_C I_C - \delta_C R_C - \epsilon_C R_C,$$

where all parameters are detailed in Table 2.
